# Clinical Practice Audit on the Management of Antineutrophil Cytoplasmic Antibody–Associated Vasculitis in the Netherlands

**DOI:** 10.1016/j.ekir.2021.08.002

**Published:** 2021-08-10

**Authors:** Ebru Dirikgil, Jacqueline T. Jonker, Sander W. Tas, Cornelis A. Verburgh, Darius Soonawala, A. Elisabeth Hak, Hilde H.F. Remmelts, Daphne IJpelaar, Gozewijn D. Laverman, Abraham Rutgers, Jacob M. van Laar, Hein J. Bernelot Moen, Peter M.J. Verhoeven, Ton J. Rabelink, Willem Jan W. Bos, Y.K. Onno Teng, Arthritis Research & Collaboration Hub (ARCH) study group

**Affiliations:** 1 Department of Nephrology, Leiden University Medical Center, Leiden, The Netherlands; 2 Department of Rheumatology and Clinical Immunology, Amsterdam University Medical Centers, location AMC, Amsterdam, The Netherlands; 3 Department of Nephrology, Spaarne Gasthuis, Haarlem, The Netherlands; 4 Department of Nephrology, Hagaziekenhuis, Den Haag, The Netherlands; 5 Department of Nephrology, Meander Medical Center, Amersfoort, The Netherlands; 6 Department of Nephrology, Groene Hart Hospital, Gouda, The Netherlands; 7 Department of Nephrology, Ziekenhuisgroep Twente, Almelo/Hengelo, The Netherlands; 8 Department of Rheumatology and Clinical Immunology, University Medical Center Groningen, Groningen, The Netherlands; 9 Department of Rheumatology and Clinical Immunology, University Medical Center Utrecht, Utrecht, The Netherlands; 10 The Dutch Vasculitis Foundation, Silvolde, The Netherlands; 11 Department of Internal Medicine, St Antoniusziekenhuis, Nieuwegein, the Netherlands

**Keywords:** antineutrophil cytoplasmic antibody–associated vasculitis, audit

## Abstract

**Introduction:**

Managing complex and rare systemic autoimmune diseases such as antineutrophil cytoplasmic antibody (ANCA)-associated vasculitis (AAV) can be challenging and is often accompanied by undesirable variations in clinical practice. Adequate understanding of clinical practice can help identify essential issues to improve the care for AAV patients. Therefore, we studied the real-life management and outcomes of AAV patients in the Netherlands.

**Methods:**

In this cohort study, we investigated clinical practice in university and nonuniversity teaching hospitals with respect to patients with a clinical diagnosis of AAV. We retrospectively collected clinical data encompassing clinical variables, medication details, and outcome parameters.

**Results:**

Data of 230 AAV patients were collected in 9 Dutch hospitals. Of these, 167 patients (73%) were diagnosed with granulomatosis with polyangiitis, 54 (24%) with microscopic polyangiitis and 9 (4%) with eosinophilic granulomatosis with polyangiitis. One hundred sixty-six patients (72%) had generalized disease. The median year of diagnosis was 2013 (range 1987–2018). Besides steroids, oral cyclophosphamide was the most used drug (50%) for induction therapy and azathioprine (68%) for maintenance therapy. Adverse outcomes were major infections in 35%, major relapses in 23%, malignancy in 10%, major cardiovascular events in 8%, and end-stage renal disease in 7%.

**Conclusion:**

Oral cyclophosphamide was the most frequently used induction therapy, azathioprine for maintenance therapy; over time, the use of rituximab is increasingly employed. Major infection and relapses are the most prevalent adverse outcomes. This audit resulted in important indicators for treatment of AAV patients that can be implemented for future, national audits to improve the outcomes of AAV patients.

## Graphical abstract

**Figure:**
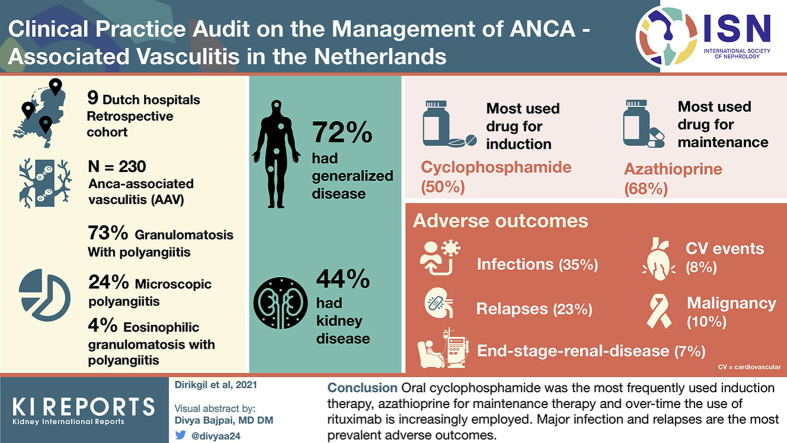

## Introduction

Antineutrophil cytoplasmic antibody (ANCA) associated vasculitis (AAV) is a rare, chronic disease often necessitating treatment with immunosuppressive medication. In the long-term AAV is associated with serious comorbidity and even mortality, which is related to disease progression, chronic damage, and treatment side effects.^1^ Practice variation is observed by patient organizations such as the Dutch Vasculitis Foundation, the members of which discuss practice variations among physicians and hospitals, reflected in divergent treatment choices. These unsolicited notions of practice variation were corroborated by audits of a relevant number of hospitals and/or physicians in the United Kingdom.^2^ However, practice variation is not necessarily a negative reflection of care delivery. Therefore, investigating practice variation is not a goal in itself but a means to detect potential gaps between scientific advancements reflected in guideline recommendations and their implementation into routine clinical practices.

In the past decade, several pivotal clinical trials in AAV have been conducted that successfully influenced treatment paradigms. Most notably, the introduction of rituximab (RTX) in remission-induction as well as remission-maintenance therapies, the position of plasma exchange, and the reduction of cumulative steroid dosing.^3^, ^4^, ^5^, ^6^, ^7^ Subsequently, recent guideline recommendations have been issued by European Renal Association – European Dialysis and Transplant Association / European Alliance of Associations for Rheumatology, British Society for Rheumatology, and Kidney Disease: Improving Global Outcomes to help and support physicians to provide the optimal treatment strategy for AAV patients.^8^, ^9^, ^10^ It is, however, well known that implementation of guideline recommendations takes time and depends on local and national health care system financing and management.^11^ Implementation strategies for guidelines are rarely investigated or audited for AAV and therefore underrepresented in the literature.

In the Netherlands, the Arthritis Research and Collaboration Hub (ARCH) was founded in 2016 with the goal of improving health care for patients with rare systemic autoimmune diseases by creating knowledge networks of hospitals in the Netherlands to share and exchange their expertise on these diseases. ARCH consists of a group of dedicated physicians with special interest and expertise in the field of systemic autoimmune diseases and members from Dutch patient organizations. For patients with AAV, ARCH set out to identify ways to improve care and outcomes for AAV patients by auditing practice variation in the Netherlands. To do so, the current study piloted an audit of the real-life practice for AAV patients in the Netherlands, focusing on treatment regimens and relevant outcomes.

## Patients and Methods

### Hospital and Patient Selection

In a national consensus conference for physicians with a special interest in AAV and in a nationwide online survey for clinicians affiliated with the Dutch Federation for Nephrology (Nederlandse Federatie voor Nefrologie, 424 physicians) and Dutch Society for Rheumatology (Nederlandse Vereniging voor Reumatologie, 395 physicians), physicians were invited to participate in this study. Participating hospitals required local approval for conducting this retrospective, cohort pilot audit in patients with a clinical diagnosis of granulomatosis with polyangiitis (GPA), microscopic polyangiitis (MPA), or eosinophilic granulomatosis with polyangiitis (eGPA). Outpatient clinics of the selected hospitals either provided a list/registry of known AAV patients (150 patients), and if this list was not present, the outpatient clinic was screened by “diagnosis registration” (80 patients). We allowed inclusion of patients who were ambulatory at the outpatient clinic in the previous 10 years (2008–2018), which coincided with the nationwide introduction of electronic health records in the Dutch health care system. Central ethical approval was obtained for the study from the medical ethics committee of the Leiden University Medical Center.

### Data Collection

Data relevant to practice variation were collected retrospectively from the time of diagnosis up to a patient’s death or maximum October 2018. Data encompassed clinical variables, medication details, and clinical outcomes. Symptoms at the time of first presentation in a hospital are categorized in the following organ systems: constitutional (myalgia, arthralgia/arthritis, fever, weight loss), cutaneous, mucous membranes/eyes, ear-nose-throat, pulmonary, cardiovascular, abdominal, renal, nervous system, and other.^12^ Disease presentation was categorized as “generalized,” defined as organ-threatening disease or involvement of 1 of the following major organs: kidneys, lungs, heart and nervous system, whereas involvement of other organ systems were called “nongeneralized” disease.^13^ For the clinical outcomes, we identified the presence or absence of any infection requiring antibiotic or antiviral therapy (including the necessity for hospitalization), any biopsy-proven malignancies, any relapse requiring (change of) therapy, major cardiovascular events (MACE; defined as myocardial infarction, cerebral infarction or hemorrhage or amputation), and end-stage renal disease (ESRD). All clinical or disease specific definitions were physician reported, and the audit did not allow for interpretation of the collected data.

### Statistical Analysis

Descriptive statistics were used to summarize patient characteristics. Numerical data were expressed as median (interquartile range [IQR]) and categorical as number (percentage). The outcomes were evaluated in a time-dependent manner using the Kaplan-Meier technique. Missing data were censored in all analyses. All analyses were performed with IBM SPSS statistics version 25 and graphs were designed with GraphPad Prism 8.0 or IBM SPSS statistics version 25.

## Results

### Study Population

We included a total of 230 AAV patients: 120 patients (52%) from 6 nonuniversity teaching hospitals and 110 patients (48%) from 3 university hospitals (Supplementary Figure S1). The median year of diagnosis was 2013 (range 1987–2018) with a median follow-up duration of 58 months (IQR 22–117 months). To verify the validity of our sample survey, we compared it with the hospital affiliations of the treating physicians from 957 members of the Dutch Vasculitis Foundation: 40% was managed in university hospitals, 41% in nonuniversity teaching hospitals, and 19% in peripheral/local hospitals (Supplementary Figure S2).

Patients’ characteristics are summarized in Table 1. Briefly, our cohort mainly consisted of Caucasian patients (95%); 101 (44%) were female and 129 (56%) male. The median age was 61 years (IQR 49–69) at the time of diagnosis. One hundred sixty-seven patients (73%) had GPA, 54 patients (24%) presented with MPA, and 9 patients (4%) with eGPA. Autoantibodies against proteinase-3 (anti-PR3) were present in 139 patients (60%) and 76 patients (33%) had autoantibodies against myeloperoxidase (anti-MPO). Eight patients (4%) had no autoantibodies, and in 6 patients (3%), no high-sensitivity autoantibody test was performed.

**Table 1:** Patient characteristics

	Total, *n* (%)
Gender	
Male	129 (56)
Female	101 (44)
Ethnicity	
Caucasian	218 (95)
Asian	1 (0.4)
Other	11 (5)
Age at diagnosis, median (IQR)	61 (49–69)
Clinical diagnosis	
GPA	167 (73)
MPA	54 (24)
eGPA	9 (4)
ANCA at diagnosis	
Anti-PR3	139 (60)
Anti-MPO	76 (33)
Both neg	8 (4)
Unknown	6 (3)
Immunofluorescence	
c-ANCA	122 (53)
p-ANCA	54 (24)
No immunofluorescence	45 (20)
Both negative	8 (4)
Symptoms at presentation in hospital	
Constitutional	122 (53)
Cutaneous	21 (9)
Mucous membranes/Eyes	20 (9)
Ear-nose-throat	99 (43)
Pulmonary	79 (34)
Cardiovascular	4 (2)
Abdominal	9 (4)
Renal	101 (44)
Nervous system	33 (14)
Other	5 (2)
Generalized	166 (72)
Nongeneralized	64 (28)

c-ANCA = cytoplasmic antineutrophil cytoplasmic antibody; eGPA = eosinophilic granulomatosis with polyangiitis; GPA = granulomatosis with polyangiitis; IQR = interquartile range; MPA = microscopic polyangiitis; MPO = myeloperoxidase; p-ANCA = perinuclear antineutrophil cytoplasmic antibody; PR3 = proteinase-3.

### Disease Characteristics

Of all patients, 166 patients (72%) had generalized disease, including 101 patients (44%) with renal, 79 patients (34%) with pulmonary, 33 patients (14%) with nervous system, and 4 patients (2%) with cardiovascular symptoms. Constitutional symptoms were present in 122 patients (53%). Furthermore, 99 patients (43%) had ear-nose-throat, 21 patients (9%) had skin, 20 patients (9%) had mucous membranes/eyes, and 9 patients (4%) abdominal symptoms (Table 1).

### Treatment Characteristics

#### Induction Therapy

**Figure 1:**
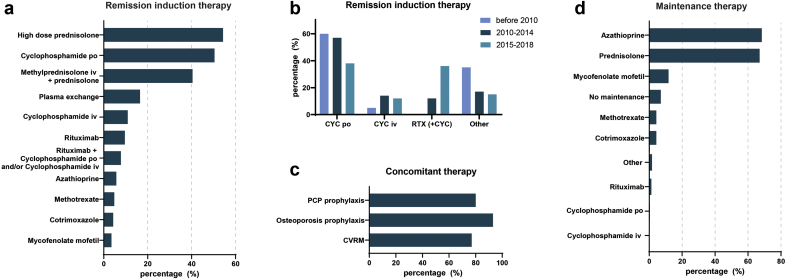
Treatment in antineutrophil cytoplasmic antibody–associated vasculitis (AAV). (a) Proportion of patients receiving remission induction therapy. (b) Proportion of patients receiving remission induction therapy divided per 5 years of start induction. Other = azathioprine, methotrexate, cotrimoxazole, or mycophenolate mofetil. Patients treated with rituximab and cyclophosphamide (combination therapy) were included in the histogram of rituximab (+cyclophosphamide); between 2010 and 2014 and 2015 and 2018, 8 of 10 patients and 10 of 30 patients were treated with combination therapy, respectively. (c) Proportion of patients receiving concomitant treatment. (d) Proportion of patients receiving any of the mentioned maintenance therapies. CVRM = cardiovascular risk management; CYC = cyclophosphamide; PCP = pneumocystis pneumonia; RTX = rituximab.

Figure 1a summarizes all agents and interventions employed as induction therapy at the time of first diagnosis. Of note, 218 patients (95%) were treated with corticosteroids (oral or i.v.) as part of the remission induction therapy. In addition to corticosteroids, 116 patients (50%) were treated with oral cyclophosphamide (CYC), 25 patients (11%) with i.v. CYC, 22 patients (10%) with RTX, and 18 patients (8%) with a combination of oral/i.v. CYC and RTX; 38 patients (17%) also required plasma exchange therapy. Other agents (azathioprine, methotrexate, cotrimoxazole, mycophenolate mofetil) were prescribed to a lesser extent. Compared with the period before 2010, we observed that after 2010, a gradual increase in the use of RTX at the expense of oral CYC use (Figure 1b). As concomitant therapy, the majority of patients received pneumocystis pneumonia prophylaxis, osteoporosis prophylaxis, and cardiovascular risk management (Figure 1c).

#### Maintenance Therapy

Figure 1d summarizes all agents prescribed as maintenance therapy. Corticosteroids were continued during maintenance in 154 patients (67%) and mainly combined with azathioprine in 157 patients (68%). Other agents such as mycophenolate mofetil and methotrexate were less commonly used, in 27 patients (12%) and 10 patients (4%), respectively. Only 1 patient was treated with long-term oral CYC and 3 patients (1%) with repeated RTX. Sixteen patients (7%) did not receive any medication for maintenance.

### Long-Term Clinical Outcomes

Table 2 summarizes relevant clinical outcomes, including infections, malignancies, disease relapses, MACE, and renal outcomes, which are addressed in more detail later in the article.

**Table 2:** Outcomes

	Patients, *n* (%)	Events, *n* (%)
Hospitalization for infection	80 (35)	158 (100)
Respiratory	52 (23)	77 (49)
Urinary	15 (7)	33 (21)
Dermatological	2 (0.9)	4 (3)
Bacteriemia	12 (5)	17 (11)
Ear-nose-throat	9 (4)	11 (7)
Gastrointestinal	8 (3)	8 (5)
Eyes	0 (0)	0 (0)
Other	3 (1)	3 (2)
E causa ignota	6 (3)	8 (5)
Malignancy	23 (10)	37 (100)
Nonmelanoma skin malignancies	14 (6)	26 (70)
Solid malignancies^a^	10 (4)	11 (30)
Relapse	91 (40)	164 (100)
Major relapse	53 (23)	102 (62)
Minor relapse	38 (17)	62 (38)
Major cardiovascular event	18 (8)	18 (100)
Myocardial infarction	10 (4)	10 (4)
Cerebral infarction	3 (1)	3 (1)
Cerebral hemorrhage	2 (1)	2 (1)
Amputation	3 (1)	3 (1)
End-stage renal disease	16 (7)	16 (7)

Outcomes in number and percentages of the total number of patients and events.

a Solid malignancies include one melanoma.

**Figure 2:**
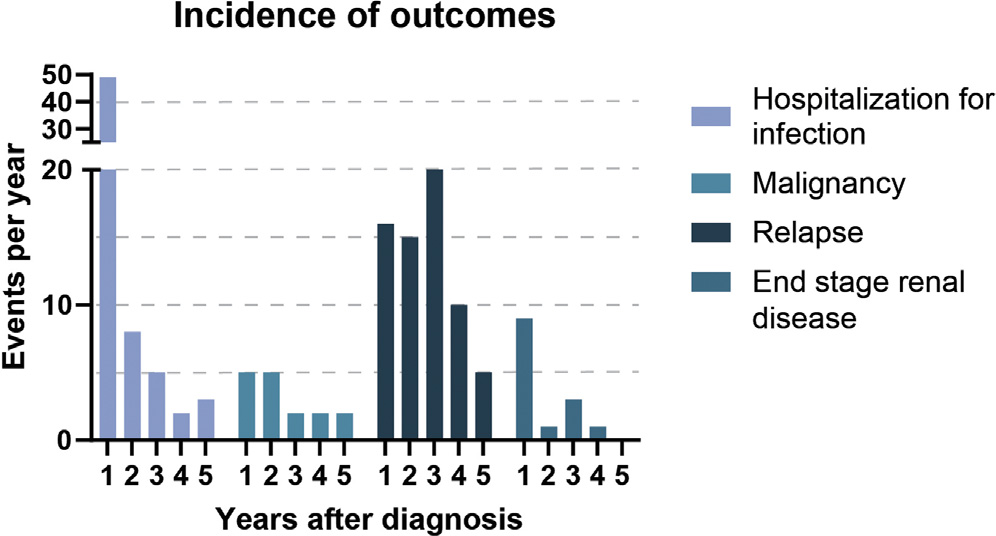
Incidence of adverse outcomes (events per year after diagnosis).

**Figure 3:**
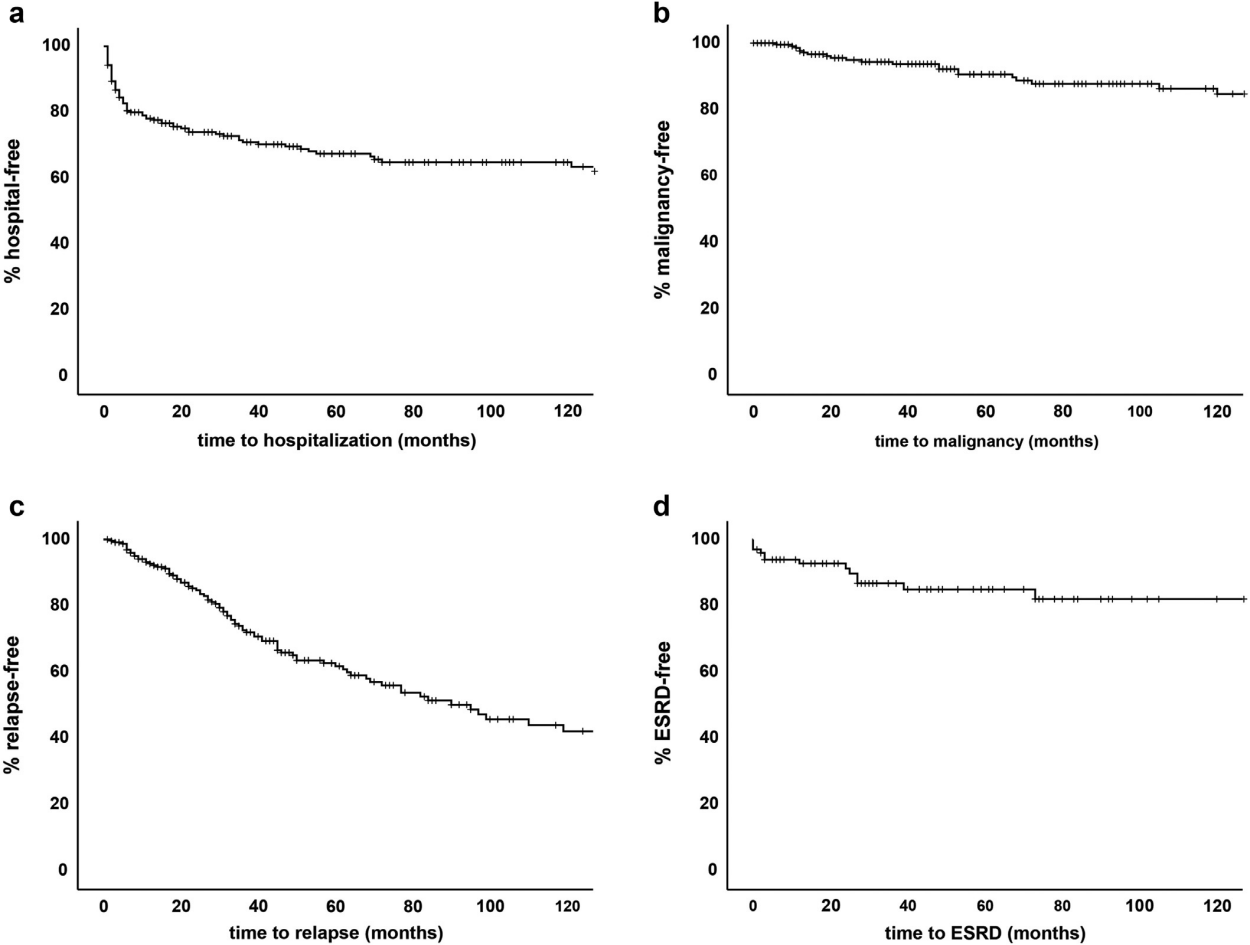
Kaplan-Meier curves over 10 years. (a) Hospitalization for infection. (b) Malignancy. (c) Relapse. (d) End-stage renal disease. Data are censored for follow-up duration.

With respect to infectious outcomes, preventive screening for infections in anticipation of immunosuppressive treatment was performed in 59 patients (26%) for tuberculosis, 68 patients (30%) for HIV, and 93 patients (40%) for hepatitis B and/or C. Nasal carriage of *Staphylococcus aureus* was tested in 93 patients (40%). During remission induction, 185 patients (80%) were treated with pneumocystis pneumonia prophylaxis. During follow-up, 100 patients (43%) had at least 1 infection, leading to hospitalization in 80 patients (35%) and death in 8 patients. In the first year after diagnosis, 49 patients were hospitalized for infection. Thereafter 8, 5, 2, and 3 patients were hospitalized for infection in the second, third, fourth, and fifth years after diagnosis, respectively (Figure 2), leading to a median time from diagnosis of AAV to hospitalization for infection of 5 months (IQR 2–30 months). Of note, 44 patients (19%) were hospitalized within the first 6 months after diagnosis (Figure 3a).

We observed 37 malignancies in 23 patients (10%). Importantly, 26 were nonmelanoma skin malignancies, and 11 were solid malignancies (including 1 melanoma). Five patients were diagnosed with a malignancy in the first year after diagnosis, and thereafter 5, 2, 2, and 2 patients were diagnosed with a malignancy in the second, third, fourth, and fifth years, respectively, after diagnosis (Figure 2), leading to a median time from AAV diagnosis to a malignancy diagnosis of 32 months (IQR 13–67 months; Figure 3b). Three patients (1%) died of malignancy.

Ninety-one patients (40%) had a total of 164 relapses. Of these, 53 patients (23%) had 102 major relapses. After diagnosis, 16, 15, and 20 patients had a relapse in the first, second, and third years, whereas 10 and 5 patients had their relapse in their fourth and fifth years (Figure 2). Cumulative relapse rates for 1 year, 3 years, and 5 years were 7%, 27%, and 38%, respectively. Median time from AAV diagnosis to relapse was median 33 months (IQR 18–64), where no statistically significant difference was observed between minor relapse (38 months; IQR 27–64) and major relapse (28 months; IQR 12–67; *P* = nonsignificant; Figure 3c).

Cardiovascular risk management therapy was prescribed to 176 patients (77%). MACE were observed in 18 patients (8%): 10 patients (4%) suffered from myocardial infarction, 3 patients (1%) cerebral infarction, 2 patients (0.9%) cerebral hemorrhage, and 3 patients (1%) an amputation.

**Figure 4:**
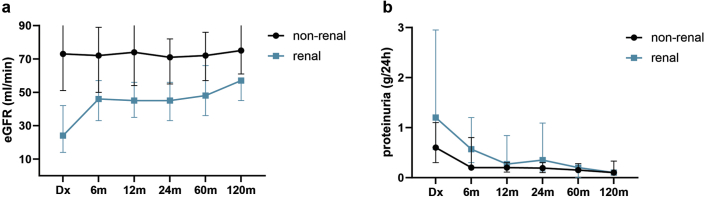
Renal function over 10 years. (a) Estimated glomerular filtration rate in milliliters per minute. (b) Proteinuria per 24 hours. Dx = time of diagnosis.

With respect to renal outcomes, kidney involvement was present in 101 AAV patients (44%) at the time of diagnosis. Sixteen patients (7% of the total population; 16% of the population with renal involvement) developed ESRD. Nine patients had developed ESRD in the first year after diagnosis; 1, 3, and 1 patients developed ESRD in the second, third, fourth, and fifth years, respectively, after diagnosis. The cumulative incidence of ESRD in patients with kidney involvement at 1 year and 5 years was 7% and 15%, respectively (Figure 3d). Overall, in AAV patients with kidney involvement, we observed an improvement of kidney function from a median 24 ml/min (IQR 15–43) to 45 ml/min (IQR 33–57) in the first 6 months after initiating remission induction therapy, after which kidney function remained fairly stable at 45 ml/min (IQR 30–60) during follow-up. Resolution of proteinuria occurred for the majority of patients after 12 months (Figure 4a and 4b).

## Discussion

The present study describes an audit of the clinical practice for AAV patients treated in nonuniversity teaching hospitals and university hospitals in the Netherlands. We identified several observations that provide support for indicators that can be addressed in future audits that can instigate improvements of care for AAV patients in the Netherlands. As such, we observed that oral CYC, besides corticosteroids, was the most prevalent choice of induction therapy, azathioprine for maintenance therapy, and the employment of rituximab is increasing. With respect to comorbidities, severe infections and disease relapses were the most common adverse outcomes for AAV patients. Together, these results provide important indicators to implement in future audits to improve the care of AAV patients in real-life clinical practice.

To evaluate the health care for AAV patients in the Netherlands, we selected AAV patients from a mix of university and nonuniversity hospitals with the aim of achieving a representative sample of patients from real-life clinical practice. To our knowledge, we present the first audit in a combination of Dutch university and nonuniversity hospitals reflecting the real-life clinical care of AAV patients. The goal of this audit study was to evaluate comprehensively how the current care is organized and identify opportunities for improvement.

If we compare the clinical practice to the most recent (inter)national recommendations for the management of AAV patients, several observations merit discussion. Our study was unable to ascertain the denominator population and report on prevalence numbers. The observed distribution of GPA, MPA, and eGPA patients were similar to the distribution of GPA, MPA, and eGPA in the rest of Europe.^14^ As induction treatment in AAV, guidelines recommend CYC or RTX for patients with generalized disease. Overall, the Dutch clinical practice is in line with these recommendation with 87% of patients with generalized disease treated with either CYC or RTX or both. The observation that half of the patients are treated with oral CYC stands out compared with guidelines that preferentially recommend i.v. CYC. According to these guidelines, the latter is based on a better safety profile than oral CYC where CYC cumulative dosage is associated with worse outcomes (e.g., malignancy and infection).^8^^,^^9^ However, in Dutch guidelines there is no clear preference between i.v. and oral CYC.^15^ In this perspective, a relevant observation of our study is that there is a transition to more frequent use of i.v. CYC and RTX in the past 5 years of the study, which may have been propelled by the approval and national reimbursement of RTX for AAV in 2015.

Regarding maintenance therapy, one needs to bear in mind that our study was conducted in 2018 when positive randomized controlled trials results for RTX as maintenance agent were not yet published.^5^, ^6^, ^7^^,^^16^ Future audits may reveal whether RTX will replace or has already replaced azathioprine as the preferred maintenance agent over the past few and in the coming years.

With respect to adverse outcomes in AAV patients, previous randomized controlled trials^3^^,^^17^ reported 11% to 12% serious infectious adverse events in the first 18 months of treatment, whereas 2 cohort studies on AAV patients treated with CYC reported higher incidence of 22% to 25% in the first 6 to 12 months.^2^^,^^18^ In long-term disease, relapses are more prevalent, with a meta-analysis reporting cumulative relapse rates of 12%, 33%, and 47% in 1, 3, and 5 years, respectively, for patients treated with CYC (oral and i.v.) as induction therapy.^19^

Our study observed 19%, 22%, and 24% hospitalization for serious infections during the first 6, 12, and 18 months and 7%, 27%, and 38% disease relapses in 1, 3, and 5 years of follow-up, respectively. As such, the outcome data from our Dutch cohort showed a trend toward a higher percentage of severe infections, whereas relapse rates seemed comparable to or even lower than international report studies investigating patients treated with CYC-based regimens. We also observed that most of the serious infections (“hospitalization for infection”) occurred in the first year after diagnosis—during the period of remission induction therapy—and that the highest relapse rates occurred in the third year after diagnosis when immunosuppressive therapy mostly is tapered or stopped. Of interest, preventive screening for opportunistic infections (such as hepatitis, tuberculosis, and HIV) before initiating remission induction therapy was performed in less than 40%, but these infections were not identified in any of the patients during the study. It is therefore of interest to evaluate how preventive infection screening can be optimized with respect to infection risks, observed rates, and cost-effectiveness.

Of interest, the prevalence of MACE was lower in our cohort than reported in the literature. MACE were observed in 8% of our Dutch cohort, which was lower than 14% reported in a recent meta-analysis,^20^ especially for ischemic heart diseases (4% vs. 35%). ESRD in our study was reported in 16%, in line with previous studies reported ESRD in 15% to 33% of patients with kidney involvement.^21^, ^22^, ^23^ Accordingly, the renal survival at 1 and 5 years was reported as 80% and 65%, respectively,^22^ whereas in our study, renal survival at 1 and 5 years was 93% and 85%. In our study, the majority (9 of 14 patients) developed ESRD in the first year after diagnosis.

It is important to mention that our study comes with several anticipated limitations. First, causal relationships between observations within a retrospective cohort study cannot be made. As such, only prospective follow-up studies that describe changes in clinical practice in detail can provide suggestions of a relation with improved adverse outcomes. Second, we conducted our study before or around the publication of several pivotal randomized controlled trials demonstrating the efficacy of RTX as a relapse-prevention strategy during maintenance.^5^, ^6^, ^7^^,^^16^ Of note, RTX was also approved and reimbursed for remission-induction in 2015 in the Netherlands. A follow-up audit will demonstrate whether clinical practice has been changed by these landmark studies. Third, this study was only able to include 9 of the 104 hospitals in the Netherlands and focused at ambulatory patients at the outpatient clinic derived from electronic health records. Consequently, our study has a high risk of immortal time bias by only including ambulant patients who were alive between 2008 and 2018. In our first design of this study, we attempted to identify all AAV patients in the Netherlands through a systematic search of electronic medical records. We were hampered by the heterogeneity/lack of uniformity of registration of AAV patients, making it practically impossible to perform the study in that ideal way. We have addressed this issue nationally because it seems a specific registration problem for systemic diseases in which multiple disciplines are involved.^24^ Although the inclusion of all centers in the Netherlands is the most ideal design for an audit, this objective is counterintuitive for a rare disease like AAV, which is mostly treated in centers with experience in managing these rare and complex patients. For this pilot audit, the present study selected 9 centers with a reasonable number of AAV patients to provide representative data and centers needed to provide ethical approval for the study and accompanying consent of patients. Therefore, the results of our study need to be interpreted taking into account these notions. Despite these limitations, we are convinced this study provides important guidance for future, nationwide follow-up audits.

In conclusion, the present study summarizes clinical practice in the care of AAV patients. This is the first study to audit treatment regimens and relevant outcomes in a real-life setting in the Netherlands. The preferred remission-induction treatment strategy (i.e., oral CYC) in all periods of this study (<2010, 2010–2014, and 2015–2018) deviates from present international guideline recommendations and is a relevant observation to discuss the implementation of novel treatment options to improve the outcome of AAV patients. It also elucidates the importance of equalizing national and international guidelines to minimalize clinical practice variation. Furthermore, data from audits and real-life practice can significantly differ from the results of landmark randomized controlled trials and demonstrates that major infection and disease relapses are the most prevalent adverse outcomes for AAV. Collectively, this study provides important guidance for future nationwide audits. Future prospects should consider an automated data extraction on relevant indicators from EMRs providing us insights into the implementation of guideline recommendations and assessing the room for improvement in the care of AAV patients.

## Disclosures

All the authors declared no competing interests.

## Supplementary Material

Supplementary File (PDF)

**Figure S1.** Participating hospitals.

**Figure S2.** Distribution of members of Dutch Vasculitis Foundation in the Netherlands.
